# Information sources and online information seeking behaviours of cancer patients in Singapore

**DOI:** 10.3332/ecancer.2018.880

**Published:** 2018-10-31

**Authors:** Gek Phin Chua, Hiang Khoon Tan, Mihir Gandhi

**Affiliations:** 1 Patient and Family Education Department, National Cancer Centre Singapore, 11 Hospital Drive, Singapore 169610, Singapore; 2 Division of Community Outreach and Philanthropy, National Cancer Centre Singapore, 11 Hospital Drive, Singapore 169610, Singapore; 3 Division of Surgical Oncology, National Cancer Centre Singapore, 11 Hospital Drive, 11 Hospital Drive, Singapore 169610, Singapore; 4 Department of Biostatistics, Singapore Clinical Research Institute, 31 Biopolis Way Nanos, #02-01, Singapore 138669, Singapore; 5 Tampere Center for Child Health Research, University of Tampere and Tampere University Hospital, Teiskontie 35, 33521 Tampere, Finland

**Keywords:** information sources, preferred sources, usefulness, cancer, oncology, Internet

## Abstract

The aim of this study was to investigate the prevalence of Internet usage among cancer patients in seeking health-related information and the type of information sought. Sources of information received from, preferences for information sources and the perceived usefulness of information from these sources were also examined in this study. A self-administered questionnaire was used to evaluate the information needs of patients undergoing cancer treatment. The questionnaire also evaluated the current source and preferred source of information as well as their online information seeking behaviours. A total of 411 patients with cancer were recruited from an ambulatory cancer centre. The patients’ physicians and healthcare specialists comprised a large majority of the patients’ information sources; they were also the most preferred source of information. 59.1% of the respondents used the Internet to search for cancer-related information, namely diagnosis and treatment options, side effects of treatment and complementary and alternative therapy; demonstrating the importance of the above information. Physicians (60.3%) and healthcare specialists (26.5%) were the largest and most preferred sources of information for cancer patients in our study. It was not uncommon for cancer patients to use the Internet to search for additional information demonstrating the need to integrate this tool more effectively for knowledge transfer for those patients who want it. It is important for healthcare professionals to help cancer patients by directing them to sources of quality information (including websites). In addition, the provision of guidelines on how to evaluate health information on the Internet would be helpful to cancer patients.

## Background

A diagnosis of cancer is a very traumatic event and patients need information to help them make treatment decisions and learn coping skills, both physically and emotionally. Good patient support in terms of information provision plays an important role in alleviating their anxiety and stress and can facilitate their decision making and enhance their coping skills and psychological well-being [1–3]. However, the type and amount of information wanted varies amongst individuals and can be influenced by the type of cancer, stage of disease, treatment, age, sex, cultural and educational background [4, 5]. Patients with cancer are also becoming active participants in their own care and information seeking has been demonstrated to play a critical role in helping patients cope with the uncertainties associated with a diagnosis of cancer and its concurrent treatment [6, 7]. Studies have indicated that patients tend to use a combination of information sources and that age, gender, education [7–12] and social economic status [8, 12–15] as well as stage of cancer at diagnosis, and their role in decision-making [2, 16] can influence patients’ information seeking behaviour. Healthcare professionals such as physicians, nurses, radiation staff and pharmacists [17–19], print information [2], others with similar illnesses [17] and websites [20–22] are the preferred sources of information.

The rapid growth of information technologies has also led to the proliferation of information available via the Internet and it follows naturally that an increasing number of patients are using the Internet to access health information [10, 14, 20, 22–25]. In Hartoonian *et al*’ [20] study, it was established that the Internet was the most preferred source of obtaining information, with twice as many survivors preferring the Internet to their healthcare providers. However, other findings indicated that healthcare professionals were the most preferred source for obtaining information [2, 7–9, 13, 17–19, 26].

In Singapore, the Internet is widely accessible as 91% of household have Internet access and 84% of individuals are Internet users [27]. The Internet may be a useful source to supplement information acquisition; however, its use is not without risk. The huge volume of information available on the Internet may overwhelm and confuse patients [8, 12, 25, 28–30]. It may also impart misleading and inaccurate information, especially from sources outside health care settings [12, 23, 28, 30]. Given substantial evidence for the importance of information in helping cancer patients with decision making [2, 3, 31] and coping [31] with their illness, an understanding of patients’ sources of information and their preference for these sources are needed. Although researchers have explored the information seeking behaviours of cancer patients including their preferred sources to obtain information, there are, however, conflicting findings and as such may not be applicable to the settings in Singapore. Furthermore, there is no reported study done in the local setting on the information sources and preferred sources among cancer patients. Knowledge of this is important to help ensure the delivery of quality cancer care by addressing patients’ information needs through preferred sources as well as directing them to sources of quality information.

In this study, we examine the prevalence of Internet use in seeking health-related information and the type of information sought by patients undergoing treatment for cancer. We also study the sources of information provision, the preferred sources and the perceived usefulness of information from these sources by these patients. Our goal is to advance the understanding of the preferred information sources and the prevalence of Internet use to facilitate the provision of patient-centred strategies to better meet the information needs of patients during cancer treatment.

## Methods

### Study conduct and analysis

All eligible patients [(1) diagnosed with cancer and receiving treatment; (2) able to understand and communicate in English/Mandarin and (3) 21 years and above] attending the ambulatory treatment unit of the National Cancer Centre Singapore during the 5-month period from 2015 to 2016 were invited to complete a 76-item survey. This survey was designed to evaluate self-reported information needs and level of satisfaction with the information received while undergoing cancer treatment; a section of the survey (36 items) was designed to assess the prevalence of Internet use in seeking cancer-related information and their sources and preferred sources of information. The questionnaire was pilot tested on 11 cancer patients and subsequently used without modification on another 411 patients. Data were collected by a research assistant who was proficient in both English and Chinese and was thoroughly briefed by the Principal Investigator regarding the data collection process including sampling and confidentiality. Consenting patients were asked to self-administer the Need Assessment Questionnaire in a language of their preference (either English or Chinese). Demographics of respondents were also collected such as race, level of education, type of diagnosis and treatment received. Sources of information were presented using frequency distribution. Association between the use of online search to receive the information and demographic characteristics were assessed using the Fisher’s exact test. A *P*-value less than 0.05 was considered statistically significant.

This study was approved by the Centralized Institutional Review Board of the Singapore Health Services.

## Results

### Response rate and characteristics of respondents

The majority of the 411 patients participating in the study were Chinese; almost two-thirds of the respondents were female, had at least secondary education and were newly diagnosed. Table 1 presents the demographics and the disease status of the full study participants.

### Sources of information and preferences

**Figure 1.**
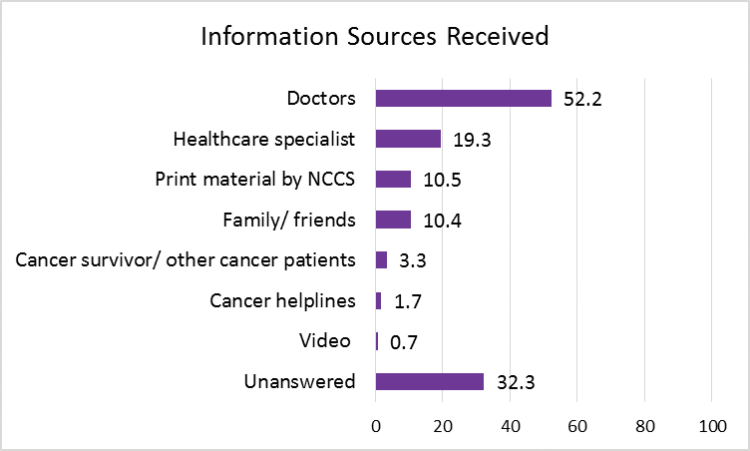
Source of information received.

**Figure 2.**
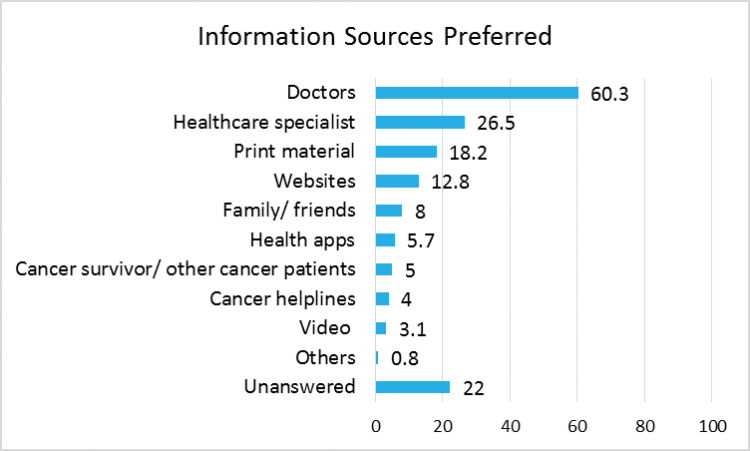
Preference of source of information to receive.

Doctors, healthcare specialists (e.g. nurses, pharmacist and radiation therapist), print material (e.g. pamphlets and brochures) from the institution and family/friends were the most common sources of information (Figure 1). When respondents were asked for their preference among a selection of sources, doctors were the most preferred, followed by healthcare specialists and print material (Figure 2). Obtaining information from websites was preferred over obtaining it from family and friends, and health apps were preferred over common information delivery systems such as others with similar illnesses, cancer helplines and videos (Figure 2).

### Usefulness of source of information received

**Figure 3.**
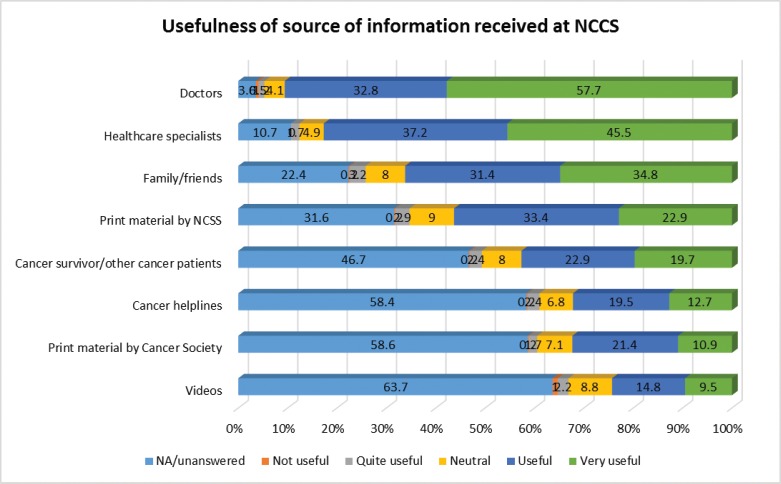
Usefulness of source of information received at the National Cancer Centre Singapore.

When asked to rate the usefulness of the information received, respondents rated information from doctors and healthcare specialist as the most useful, followed by those from family/friends (Figure 3). Although print information ranked third as the preferred source of information (Figure 2), it was ranked fourth in term of usefulness (Figure 3).

### Internet use

The study revealed that the Internet was another resource that cancer patients sought information from over the past year. More than half of the respondents had used the Internet to find information, with cancer diagnosis and treatment options being the most sought after (90%), followed by possible side effects of treatment (87%) and complementary and alternative therapy (70%). Only 43% used the Internet to find supportive resources and less than half (47%) disagreed that the information was hard to understand (Table 2). Younger patients, those of a higher education standard and female patients were found to be more likely to search for information online (Table 3).

## Discussion

### Information source and preferred source of information and usefulness of information received from various sources

Patients in our study indicated that the top three preferred sources of cancer information are a doctor, a healthcare specialist and print material, respectively. The finding that doctors and healthcare specialists are preferred information sources emphasises the crucial role that they play in meeting patients’ information needs. This pattern of information use and preference is to be expected as patients perceived these professionals to possess the expert knowledge and print material from healthcare institutions to be more reliable. Our findings support those of previous studies [2, 7–9, 13, 17–19, 31]. A study by Shea-Budgell *et al* [7] found healthcare professionals (84%) to be the most preferred source of information, followed by personalised reading material (75%). Health care professionals were also found to be the most trusted source of cancer information, including personalised written information from their healthcare provider. Hesse *et al*’ study [9] also revealed physicians to be the most highly trusted information source to patients (with 62% of adults expressing a lot of trust in their physicians). However, almost half (49%) of the patients in that study reported going online for information first, with only 11% going to their physicians first. This demonstrates the desire for rapid access to information among cancer patients.

The preference to obtain information from doctors and healthcare specialists in our study may arise from the perceived usefulness of information from these sources. The information given by the doctors and healthcare professionals were rated as useful or very useful by 90% and 83% of the patients in our study, respectively. The perceived usefulness of family and friends as information sources (66%) in our study indicates that family and friends play an important role when dealing with a complex disease, especially when the patient’s ability to process and comprehend information may be impaired due to the stress of illness [13]. This is evidenced by the findings that patient of a lower education level (primary or lower) is more likely to receive information from family and friends compared to those of a higher education level (secondary and higher). Our study also found that the level of education is not associated with the usage of print information which may infer that the level at which the information is written in such material appropriately meets their needs or that their information needs are being met by their family and friends (Table 4).

### Online information seeking behaviour and understanding health information

The complexity of cancer information, as well as the challenge in making treatment decisions, may compel patients to seek an alternative source of information. Responses in our study indicated that slightly more than half of the respondents used the Internet and that half of these Internet users used it frequently to seek cancer information during the past 1 year. The majority of patients used the Internet to seek information on cancer diagnosis and treatment options, possible side effects of treatment and complementary and alternative therapy, demonstrating the importance of the above information. In a systematic review of 112 articles, Rutten *et al* [13] established that the most frequent information need was treatment-related (38%) and that during diagnosis and treatment, information needs about the stage of disease, treatment options and side-effects of treatment were prominent. Other studies also supported these findings [3, 16, 18, 32].

Our study also indicated that both younger patients (who tend to be better educated) as well as female patients are more likely to go online to search for cancer information. This finding is similar to previous work [8, 9, 14]. In Mayer *et al*’ study [8], the researchers found that patients with cancer who use the Internet as a main source of information were typically women, under the age of 65 and with a higher level of income. Their study also revealed that the most frequently cited reasons for seeking cancer information on the Internet were convenience, more available information and immediacy of access. Decreasing age and having a higher education level were also found to be associated with patients wanting to play an active role in medical decision making [33].

The Internet has tremendous potential to empower patients and support their ability to make informed health-related decisions. However, information from the Internet is of highly variable quality and not necessarily easy to comprehend [8, 12, 23, 25, 28–30]. The lack of regulatory oversight on the posting of medical and health information on the Internet thus poses inherent risks. Some information may not be developed by healthcare professionals, evidence-based or systematically reviewed and as such, may be incomplete, inaccurate, inappropriate or erroneous [12, 23, 30]. Another risk is the resources recommended may be commercially driven and offer expensive solutions that would not otherwise be recommended by healthcare professionals. These can potentially lead to misinformed and distressed patients and an increased tendency toward self-diagnosis or self-treatment [23] or engaged in a behaviour counter to their health care needs [20, 28].

Furthermore, quality health information is ineffective if it is presented in such a way as to render it incomprehensible to the consumer. Of the 245 respondents who used the Internet in the past year, only 114 (46.6%) of the Internet users in our study found such information to be easily comprehensible. The risks of unreliable and unclear information from the Internet are supported by other studies [23, 28, 34]. In Helft *et al*’ [34] study, the researchers found that 54% of oncologists considered the Internet to have a negative effect on their patients and the patient–physician relationship as a result of increased confusion. However, their findings on patient–physician relationship contradicted with other studies [35–37]. Published studies indicate that only a small proportion of patients discussed information obtained from the Internet with their healthcare providers [7, 10, 30, 34]. Barriers to discussion of online information were concerns about embarrassment, concerns that the doctor doesn’t want to hear about it, believe that there is no need to bring it up, forgetting to bring it up [30], fear of being perceived as challenging or confronting their physician [25, 29, 35, 38, 39], respected doctors’ authority and not used to ask questions to doctors [39], and lack of time or reluctance to interfere with the consultation process [25, 29]. Facilitators to communication included having a family member present at doctor’s visit [30], doctor-initiated inquiries [30, 40] and encountering an advertisement that suggested talking with a doctor [30]. Although the effect of information obtained from the Internet on the patient–physician relationship is debatable [34–37], there is evidence that the Internet may adversely affect the decisions made by patients with cancer [29, 34]. It is therefore important for healthcare professionals to encourage patients to discuss their Internet information searches and help patients by directing them to sources of quality information on the Internet. In addition, guidelines on how to evaluate health information on the Internet would be helpful to cancer patients.

Although doctors and healthcare specialists are consistently cited as important sources of health information, continued evaluation of the sources from which cancer patients seek information is necessary. This is due to the constant evolution of information technology as well as identifying their preferences on how information is to be delivered will enable delivery systems that would best match their needs. This is also important as our study also revealed that respondents also preferred to receive information from websites and health apps in place of other common forms of information delivery systems such as cancer survivors, cancer helplines and videos. Providing patients with trusted information sources that match their informational needs may assist patients with recovery [10].

## Limitations

There are several limitations inherent in this study. First, the results were obtained from a sample of patients treated in only one institution (National Cancer Centre Singapore). It is unclear whether the results are generalisable to other patient populations and other healthcare settings in a different country. Furthermore, this report is a part of a larger study, and factors that may preclude a more complete analysis have not been included. These include, but are not limited to, the level of trust in the healthcare sources and the reasons for going or not going online for information. Nevertheless, the study provides useful baseline information to aid in the better delivery of healthcare information to cancer patients.

## Conclusions

Cancer patients need information to help them make treatment decisions and to cope with their physical and psychological needs. Our study indicates that the most preferred sources of information are from doctors, healthcare specialists and print material. It demonstrates the crucial role that physicians, healthcare specialists and print material play in meeting patients’ information needs. The preference to receive information from these sources may have arisen from the perceived usefulness of information from these sources. More than half of the respondents, however, also used the Internet to seek information on cancer diagnosis and treatment options, possible side effects of treatment and complementary and alternative therapy. Age, gender and education are predictors of online information search.

To our knowledge, the present study is the first to examine the information seeking behaviour of cancer patients undergoing treatment in Singapore (with regard to the information sources consulted, the preferences for these sources and the perceived usefulness of information from these sources).

## Practice implications

Providing cancer patients with quality information in order for them to make informed decisions and to enhance their coping skills is one of the aspects of patient-centred care. However, this can be extremely challenging during typical consultations or treatment sessions. As such, a multipronged approach with regard to information delivery based on identified preferences could be an effective strategy. As the print material was found to be another source that the patients prefer, this could be an effective supplementary source for information delivery. Patients are also using the Internet to search for cancer information and their preference to use health apps in place of other common forms of information delivery system emphasise the need for healthcare professionals to embrace these tools to enhance information delivery. Strategies to mitigate the risks of erroneous or conflicting information that may be found online could include providing patients with a list of trusted information sources that match their information needs and establishing guidelines on how to evaluate health information from the Internet. Identifying and continuing evaluation of the sources from which cancer patients seek information and their usage of these sources, and type of information sought, is necessary to ensure the information and the mode of delivery best matches their needs.

## Conflicts of interest

The authors have no financial conflicts of interest to declare.

## Funding

This research was made possible by a grant from the Community Cancer Fund [COMCF-YR2015-MAY-NS1].

## Figures and Tables

**Table 1.** Characteristics of survey respondents.

Characteristics, *n* (%)	*N* = 411
*Sex*	
Male	153 (37.0)
Female	259 (63.0)
*Age (years)*	
21–40	45 (10.9)
41–60	223 (54.3)
60 and above	142 (34.5)
Unknown	1
*Ethnicity*	
Chinese	317 (77.1)
Malay	46 (11.2)
Indian	20 (4.9)
Others	27 (6.6)
Unknown	1
*Highest level of education*	
Primary or less	81 (19.7)
Secondary/Higher Secondary	213 (51.8)
Tertiary	109 (26.5)
Unknown	8
*Type of treatment (n = 411)*	
Surgery	248 (60.3)
Radiation therapy	118 (28.7)
Chemotherapy	404 (98.3)
Hormonal therapy	45 (10.9)
Clinical trials	62 (15.1)
*Cancer type*	
Breast	115 (28.0)
Colon/rectal	60 (14.6)
Lung	52 (12.6)
Others	171 (41.6)
Unknown	39
*Disease status*	
Newly diagnosed	304 (74.0)
Recurrent	98 (23.8)
Unknown	9

**Table 2.** Usage of Internet in the past year to find information related to the disease.

Category, *n* (%)	All patients (*N* = 411)
*Usage of Internet*	
Never	162 (39.4)
Rarely	35 (8.5)
Sometimes	106 (25.3)
Often	104 (25.3)
Unknown	4
Information searched*	*n* = 245
Cancer diagnosis and treatment options	221 (90.2)
Possible side effects of your treatment	213 (86.9)
Complementary and alternative therapy	172 (70.2)
Supportive resources	105 (42.9)
Information obtained online hard to understand?*	
Strongly agree	2 (0.8)
Agree	35 (14.3)
Neutral	91 (37.1)
Disagree	94 (38.4)
Strongly disagree	20 (8.2)

* Percentages are based on patients who have used the Internet in the past year (*n* = 245)

**Table 3.** Association between demographic characteristics and online information search.

Characteristics, *n* (%)	*N*	Online information search		
		Yes (Sometimes, Often)	No (Never, Rarely)	*P*-value
*Age*				< 0.001
21–40 years	45	35 (77.8)	10 (22.2)	
41–60 years	220	136 (61.8)	84 (38.2)	
> 60 years	141	39 (27.7)	102 (72.3)	
*Sex*				0.024
Male	150	66 (44.0)	84 (56.0)	
Female	257	144 (56.0)	113 (44.0)	
*Education level*				< 0.001
Primary or less	80	14 (17.5)	66 (82.5)	
Secondary/higher secondary	210	109 (51.9)	101 (48.1)	
Tertiary or higher	109	83 (76.1)	26 (23.9)	

**Table 4.** Association between demographic characteristics and current and preferred source of information

Characteristics, *n* (%)	*N*	Doctors		Healthcare specialist		Written material		Family or friends	
		Current	Preferred	Current	Preferred	Current	Preferred	Current	Preferred
*Age*									
21–40 years	45	44 (97.8)	42 (93.3)	27 (60.0)	26 (57.8)	18 (40.0)	15 (33.3)	18 (40.0)	7 (15.6)
41–60 years	223	210 (94.2)	200 (89.7)	114 (51.1)	118 (52.9)	69 (30.9)	82 (36.8)	62 (27.8)	40 (17.9)
> 60 years	142	136 (95.8)	128 (90.1)	68 (47.9)	57 (40.1)	30 (21.1)	28 (19.7)	38 (26.8)	32 (22.5)
*P*-value of association		0.672	0.860	0.360	0.027	0.024	0.002	0.210	0.452
*Sex*									
Male	152	144 (94.7)	133 (87.5)	63 (41.4)	64 (42.1)	32 (21.1)	37 (24.3)	36 (23.7)	22 (14.5)
Female	259	247 (95.7)	238 (91.9)	146 (56.4)	138 (53.3)	85 (32.8)	88 (34.0)	83 (32.0)	58 (22.4)
*P*-value of association		0.814	0.169	0.004	0.032	0.013	0.046	0.073	0.054
*Education level*									
Primary or less	81	77 (95.1)	68 (84.0)	49 (60.5)	41 (50.6)	21 (25.9)	16 (19.8)	33 (40.7)	29 (35.8)
Secondary/higher secondary	213	201 (94.4)	192 (90.1)	97 (45.5)	94 (44.1)	56 (26.3)	56 (26.3)	55 (25.8)	31 (14.6)
Tertiary or higher	109	105 (96.3)	103 (94.5)	58 (53.2)	63 (57.8)	36 (33.0)	50 (45.9)	27 (24.8)	17 (15.6)
*P*-value of association		0.786	0.057	0.058	0.065	0.404	< 0.001	0.028	< 0.001

## References

[1] Lang EV, Berbaum KS, Lutgendorf SK (2009). Large-core breast biopsy: abnormal salivary cortisol profiles associated with uncertainty of diagnosis. Radiology.

[2] Davison BJ, Breckon EN (2012). Impact of health information-seeking behaviour and personal factors on preferred role in treatment decision making in men with newly diagnosed prostate cancer. Cancer Nurs.

[3] Lo AC, Olson R, Feldman-Stewart D (2015). A patient-centered approach to evaluate the information needs of women with ductal carcinoma in situ. Am J Clin Oncol.

[4] Bernstein KI, Promislow S, Carr R (2011). Information needs and preferences of recently diagnosed patients with inflammatory bowel disease. Inflamm Bowel Dis.

[5] Rolnick SJ, Altschuler A, Nekhlyudov L (2007). What women wish they knew before prophylactic mastectomy. Cancer Nurs.

[6] Liu JE, Mok E, Wong T (2005). Perceptions of supportive communication in Chinese patients with cancer: Experiences and expectations. J Adv Nurs.

[7] Shea–Budgell MA, Kostaras X, Myhill KP (2014). Information needs and sources of information for patients during cancer follow-up. Curr Oncol.

[8] Mayer DK, Terrin NC, Kreps GL (2007). Cancer survivors information seeking behaviors: a comparison of survivors who do and do not seek information about cancer. Patient Educ Couns.

[9] Hesse BW, Nelson DE, Kreps GL (2005). Trust and sources of health information. The impact of the internet and its implications for health care providers: Findings from the first health information national trends survey. Arch Intern Med.

[10] Eheman CR, Berkowitz Z, Lee J (2009). Information-seeking styles among cancer patients before and after treatment by demographics and use of information sources. J Health Commun.

[11] Rutten LJF, Squiers L, Treiman K (2006). Requests for information by family and friends of cancer patients calling the national cancer institute’s cancer information service. Psychoncology.

[12] Zilinski L (2010). Information behaviours of cancer patients in the information age. Library Student Journal.

[13] Rutten LJF, Arora NK, Bakos AD (2005). Information needs and sources of information among cancer patients: a systematic review of research (1980–2003). Patient Educ Couns.

[14] Jacobs W, Amuta AO, Jeon KC (2017). Health information seeking in the digital age: an analysis of health information seeking behaviour among US adults. Cogent Social Science.

[15] Ramanadhan S, Viswanath K (2009). Health and the information nonseeker: a profile. Health Communication.

[16] Lambert SD, Loiselle CG, Macdonald ME (2009). An in-depth exploration of information-seeking behavior among individuals with cancer—Part 1: understanding differential patterns of active information seeking. Cancer Nurs.

[17] Lambert SD, Loiselle CG, Macdonald ME (2009). An in-depth exploration of information-seeking behavior among individuals with cancer—Part 2: understanding patterns of information disinterest and avoidance. Cancer Nurs.

[18] Li PWC, So WKW, Fong DYT (2011). The information needs of breast cancer patients in Hong Kong and their levels of satisfaction with the provision of information. Cancer Nurs.

[19] Miyashita M, Ohno S, Kataoka A (2014). Unmet information needs and quality of life in young breast cancer survivors in Japan. Cancer Nurs.

[20] Hartoonian N, Ormseth SR, Hanson ER (2014). Information-seeking in cancer survivors: application of the comprehensive model of information seeking to HINTS 2007 Data. J Health Commun.

[21] Spector D, Mayer DK, Knafl K (2010). Not what I expected: informational needs of women undergoing breast surgery. Plast Surg Nurs.

[22] Roach AR, Lykins ELB, Gochett CG (2009). Differences in cancer information seeking behaviour, preferences and awareness between cancer survivors and healthy controls: a national, population-based survey. J Cancer Educ.

[23] Huang GJ, Penson DF (2008). Internet health resources and the cancer patient. Cancer Invest.

[24] Knijnenburg SL, Kremer LC, van den Bos C (2010). Health information needs of childhood cancer survivors and their family. Pediatr Blood Cancer.

[25] Lopez-Gomez M, Ortega C, Suarez I (2012). Internet use by cancer patients: should oncologists ‘prescribe’ accurate web sites in combination with chemotherapy? A survey in a Spanish cohort. Ann Oncol.

[26] Ankem K (2006). Use of information sources by cancer patients: results of a systematic review of the research literature. Inform Res.

[27] Infocomm Media Development Authority (IMDA) (2018). Infocomm usage—households and individual [Internet].

[28] Ahmad F, Hudak PL, Bercovitz K (2006). Are physicians ready for patients with internet-based health information?. J Med Internet Res.

[29] Sommerhalder K, Abraham A, Zufferey MC (2009). Internet information and medical consultations: experiences from patients’ and physicians’ perspectives. Patient Educ Couns.

[30] Silver MP (2015). Patient perspectives on online health information and communication with doctors: a qualitative study of patients 50 years old and over. J Med Internet Res.

[31] Hesse BW, Arora NK, Beckjord EB (2008). Information support for cancer survivors. Cancer.

[32] Maddock C, Lewis I, Ahmad K (2011). Online information needs of cancer patients and their organizations. ecancer.

[33] Say R, Murtagh M, Thomson R (2006). Patients’ preference for involvement in medical decision making: a narrative review. Patient Educ Couns.

[34] Helft PR, Hlubocky F, Daugherty CK (2003). American oncologists’ views of Internet use by cancer patients: a mail survey of American Society of Clinical Oncology members. J Clin Oncol.

[35] Schrank B, Sibitz I, Unger A (2010). How patients with schizophrenia use the internet: qualitative study. J Med Internet Res.

[36] Newnham GM, Burns WI, Snyder RD (2006). Information from the internet: attitudes of Australian oncology patients. Intern Med J.

[37] AlGhamdi KM, Moussa NA (2012). Internet use by the public to search for health-related information. Int J Med Inform.

[38] Hay MC, Cadigan RJ, Khanna D (2008). Prepared patients: internet information seeking by new rheumatology patients. Arthritis Rheum.

[39] Chiu Y (2011). Probing, impelling but not offending doctors: the role of the internet as an information source for patients’ interactions with doctors. Qual Health Res.

[40] Stevenson FA, Kerr C, Murray E (2007). Information from the internet and the doctor-patient relationship: the patient perspective—a qualitative study. BMC Fam Pract.

